# Social support, physical activity and sedentary behavior among 6^th^-grade girls: a cross-sectional study

**DOI:** 10.1186/1479-5868-3-8

**Published:** 2006-04-06

**Authors:** Andrew E Springer, Steven H Kelder, Deanna M Hoelscher

**Affiliations:** 1 Center for Health for Health Promotion and Prevention Research, School of Public Health, The University of Texas Health Science Center at Houston, Houston, Texas, USA; 2 Human Nutrition Center, School of Public Health, The University of Texas Health Science Center at Houston, Houston, Texas, USA

## Abstract

**Background:**

Despite the importance of social support in promoting physical activity, little is known about the relative influence of the type or source of social support on adolescent girls' physical activity and sedentary behaviors. This study examined the associations of two types of social support (social participation in and social encouragement for physical activity) and two social support sources (family and friends) with self-reported daily minutes of physical activity and sedentary behavior among sixth-grade girls in Texas.

**Methods:**

A secondary analysis of 718 sixth-grade girls between the ages of 10 to 14 was performed using cross-sectional baseline data from an osteoporosis prevention intervention study. Physical activity and sedentary behaviors (television-video viewing and computer-video game playing) were assessed using 3 administrations of the Self-Administered Physical Activity Checklist; social support indicators were assessed with Likert-type items from a psychosocial questionnaire.

**Results:**

In multiple linear regression analyses, friend physical activity participation (partial correlation coefficient (r) = 0.10, p = .009) and friend (r = 0.12) and family encouragement (r = 0.11) (p < .01, respectively) were positively related to moderate-to-vigorous physical activity in the full model with other support variables, BMI and ethnicity; friend encouragement was the only variable positively related to vigorous physical activity (r = 0.11, p = .005). Family participation in physical activity had the strongest negative correlation with total minutes of television-video viewing and computer-video playing (r = -0.08, p < .05).

**Conclusion:**

Findings lend support to the importance of social support for physical activity among adolescent girls but suggest that the source and type of social support may differ for physical activity and sedentary behaviors. Further research is needed to assess the causal or reciprocal relation between the roles of friends and family in promoting physical activity and of family physical activity in decreasing sedentary behaviors among early adolescent girls.

## Background

Regular physical activity is associated with a variety of health benefits, including a reduced risk for heart attack, colon cancer, diabetes, high blood pressure and possibly stroke [1]. Despite the importance of establishing a physically active lifestyle early in life [2], results from a CDC national study of children aged 9 to 13 indicated that 61.5% do not participate in any organized physical activity during their non-school hours and that 22.6% do not engage in any free-time physical activity [3]. Children tend to become less physically active as they move through adolescence, and female adolescents have been found to engage in less physical activity than male adolescents [4,5].

Sedentary behaviors such as television watching have been associated with potentially adverse health conditions such as child overweight [6-8] and have been hypothesized to displace time spent in physical activity [9,10]. Two common sedentary behaviors among children are television watching and computer video game playing, with one national study indicating that U.S. children view an average of 199 minutes of television and video and play 33 minutes of video games per day [11]. The health consequences of inactivity, the large proportion of U.S. children who are inactive, and gender prevalence disparities in physical activity underscore the need for continued exploration of the factors that promote physical activity among adolescents, especially among females.

Physical activity is a complex behavior influenced by multiple factors within the environmental, social/cultural, and psychological/cognitive domains [12]. Within the social/cultural domain, social support has been associated with engagement in regular physical activity among young people [5]. Social support for physical activity has been conceptualized using various typologies [13,14], including instrumental and direct support (e.g., transportation to an exercise class); emotional and motivational support (e.g., encouragement, praise); and observational support (e.g., modeling of healthy behaviors). Although public health researchers have recommended additional research on the type of social support most conducive to physical activity in children and adolescents [15], few studies have examined the relative influence of a specific type of social support on adolescent physical activity.

Similarly, while it is generally accepted that family, peers, and school influence physical activity in children [16], few studies have examined the relative influence of different sources of social support on physical activity levels of adolescents. Developmental theory posits that family influences decrease while peer influences increase as children pass through puberty [17,18]. Although some studies have found peer influence to be an important predictor of physical activity in middle school students [13,15], Sallis et al. [15] and Silver et al. [19] found family influence to be a stronger predictor among female high school and college students, respectively – findings contrary to developmental theory. Further research is needed to better understand the relative influence of peer and parental/family influences on physical activity among adolescents.

The current study explores the associations between different types and sources of social support and physical activity among adolescent girls. Specifically, this study examined the associations of two types of social support (social participation and social encouragement) and two sources of social support (from friends and family) with moderate and vigorous physical activity and with the sedentary behaviors of television-video viewing and computer-video game playing. In contributing to the body of literature on childhood obesity, we also explored the associations between body mass index (BMI) and social support and BMI and physical activity/sedentary behavior.

## Methods

### Sample and procedures

This study is based on a secondary analysis of baseline data from the IMPACT Study (Incorporating More Physical Activity and Calcium in Teens), a two-year, school-based health education intervention aimed at increasing bone accretion by promoting calcium containing foods and physical activity in middle school girls. The study participants were recruited from 12 public middle schools located in a suburban area of a large city in central Texas. The final sample included in the baseline study was 718 female students. Participants were primarily white (72%), attended sixth grade, and had a mean (SD) age of 11.6 (.39) years (Table 1). Parental consent and student assent were obtained before participation in the measurements. The study was approved by the Human Subjects Review Committees at the University of Texas-Houston, School of Public Health, Baylor College of Medicine, and school district research committees. Further details on study design and methods are described elsewhere [20].

**Table 1:** Descriptive characteristics and aggregate mean scores of daily minutes of physical activity and sedentary behaviors among female middle school students, IMPACT Baseline Study, 2001 (n = 718)

Descriptor	Total Sample		Moderate-Vigorous PA min.		Vigorous PA min.		TV-video viewing min.		Computer-video game playing min.		Combined sedentary min.^c^	
						
	n	%	mean	SD	mean	SD	mean	SD	mean	SD	mean	SD
Total Sample	718	100	65.8	57.8	17.6	34.1	102.4	86.8	22.4	36.2	124.8	98.3
												
Ethnicity												
White (non-Hispanic)	515	71.8	68.8	54.2	15.7	29.5	96.0	83.8	21.0	32.4	117.0	94.2
Hispanic	83	11.6	75.6	63.3	22.3	36.2	127.5	87.3	18.2	40.8	145.7	95.7
Other^a^	119	16.6	68.0	60.4	22.9	40.1	113.7	95.1	31.0	46.4	144.7	112.5
			p = .607		p = .042		p = .003		p = .014		p = .003	
												
BMI-for-age percentiles^b^												
5th percentile	28	3.9	51.9	41.5	11.1	22.6	84.1	83.7	21.5	28.9	105.6	89.8
>5th to <85th percentile	510	71.0	71.6	57.9	17.3	32.1	97.0	84.1	20.5	33.5	117.5	95.4
85th to <95th percentile	105	14.6	67.2	53.9	17.5	29.1	105.2	79.1	22.6	41.3	127.8	89.6
95th percentile	74	10.3	63.9	53.0	22.6	40.9	140.2	104.8	34.3	46.1	174.5	115.5
			p = .247		p = .424		p = .001		p = .025		p < .001	

Abbreviations: PA, physical activity; min., minutes; SD, standard deviation,

^a^Includes African American (n = 39), Asian/Pacific Islander (n = 26), Native American (n = 7), and other (n = 47)

^b^Calculations and classifications based on National Center for Health Statistics and National Center for Chronic Disease Prevention and Health Promotion growth charts, with 5th percentile = underweight; >5th to <85th percentile = normal weight; 85th to <95th percentile = at risk for overweight; and 95th percentile = overweight

^c^Combined Sedentary Behavior is the sum of daily T.V.-video viewing minutes and daily computer-video game playing minutes

### Description of measures

Study variables included physical activity, sedentary behaviors, two indicators of social support, body mass index (BMI), and ethnicity.

Physical activity was measured in daily minutes using a physical activity checklist. The Self-Administered Physical Activity Checklist (SAPAC), developed and validated with a multi-ethnic 5^th ^grade sample, is intended to assess intensity (e.g., moderate or vigorous), duration, and types of physical activity [21]. The SAPAC is a one-day recall of 22 common physical activities that also collects time spent watching television and playing video games. The self-report version was validated against heart rate monitors (r = 0.57, p = .0001), Caltrac accelerometers (r = 0.30, p = .001), and interviewer-administered checklists, (r = 0.76, p = .0001) in 125 fifth-grade children [21]. To improve precision, the SAPAC was administered to study participants on three separate days, which included one weekend day and two weekdays, and an average score was calculated for each participant. This study included moderate-vigorous physical activity (MVPA), which is the sum of minutes of activities corresponding to these intensity levels, and vigorous physical activity (VPA).

Sedentary behaviors were measured using three variables from the SAPAC: a.) mean daily minutes of television-video viewing; b.) mean daily minutes of computer-video game playing; and c.) mean daily minutes of combined sedentary behavior (the sum of television-video viewing and computer-video game playing minutes). All of the sedentary variables represented the mean values from the three days of SAPAC measurements.

Four items assessed social support by asking students to rate how often during the past month their: a.) family did physical activities with them; b.) family encouraged them to be physically active; c.) friends did physical activities with them; and d.) friends encouraged them to be physically active. All items used a five-point Likert scale that ranged from 'never' to 'most of the time'. These items come from the Calcium Osteoporosis Physical Activity (COPA) Questionnaire, an 85-item self-administered questionnaire developed specifically for the IMPACT study that assesses behavioral and psychosocial aspects of physical activity and nutrition. These items were adapted from similar questions used in the Child and Adolescent Trial for Cardiovascular Health [22], which had been validated and were shown to detect treatment-control differences after one [22] and two years of intervention [23].

BMI was included as a covariate based on its association with physical activity [5] and sedentary behavior such as television watching [6,8]. BMI is calculated as weight in relation to height (kg/meters^2^). Weight was measured using a top-loading digital scale (SECA 770 or Tanita BWB-800S), and height was measured with standard stadiometers (Perspectives Enterprises). Calibration weights were used to calibrate the scales up to 200 lbs. before each set of measurements.

Study participants self-described their ethnicity according to pre-determined ethnic classifications as used in previous work by the investigators (24, 25).

### Analysis

Data were analyzed using SPSS 13 (SPSS Inc., Chicago, Ill). Descriptive statistics were initially calculated to describe the sample and the distribution of the variables. Daily physical activity minutes and combined sedentary minutes were treated as continuous variables. In describing the sample, a one-way analysis of variance (ANOVA) was conducted to evaluate mean differences of the physical activity and sedentary behavior daily minutes by ethnicity and BMI-for-age percentile categories. Pearson correlation coefficients were computed to evaluate the bivariate relation between the social support variables and the physical activity and sedentary behavior outcome variables. Lastly, separate multiple linear regression analyses were conducted to predict each criterion variable (MVPA minutes, VPA minutes, or combined sedentary minutes) from the four social support variables, with BMI and ethnicity included as covariates. Significant differences at or below the .05 level in physical activity or sedentary minutes by ethnic group and BMI-for-age categories provided a basis for inclusion of these covariates in the multivariable model. In carrying out the analyses, we used a simultaneous regression analysis approach that included the same set of covariates in order to allow for comparability among the physical activity/sedentary behavior outcome variables. Pearson correlation coefficients were calculated to determine the level of collinearity between the social support variables. In evaluating the appropriateness of the use of linear regression analysis, the General Linear Model function was used to compare the bivariate results of the relation between the independent and dependent variables using a model assuming linearity (regression) with the results using a model not assuming linearity (ANOVA). Differences were considered statistically significant if the p value was < .05.

## Results

Students participating in the study at baseline reported a mean (SD) of 65.8 (57.8) minutes of daily MVPA and 17.6 (34.1) minutes of daily VPA (Table 1). Students reported a higher number of minutes of sedentary behavior, with a mean of 124.8 (98.3) combined sedentary minutes per day. Although BMI was not associated with physical activity, it was positively associated with TV-video viewing (p = .001) and computer-video game playing (p = .025). Lastly, BMI was not associated with any of the four social support variables [data not shown].

We found no significant differences between the ANOVA model (not assuming linearity) and the linear regression model (assuming linearity) for the vigorous physical activity and combined sedentary behavior outcome variables, which provided support for the use of linear regression methods to analyze these variables. However, significant differences between the two models were found for the relation between family encouragement and MVPA (p < .05), indicating the need for an alternate strategy. Based on these results, a quadratic form of the family encouragement variable – created by centering and squaring the variable – was subsequently included in the full model.

In assessing the collinearity of the social support variables for the multiple linear regression analysis, Pearson correlation coefficients indicated a low to moderate correlation, with coefficients ranging from r = .24 for family encouragement and friend participation to r = .47 for friend participation and friend encouragement [data not shown].

### Moderate-Vigorous Physical Activity (MVPA)

**Figure 1:**
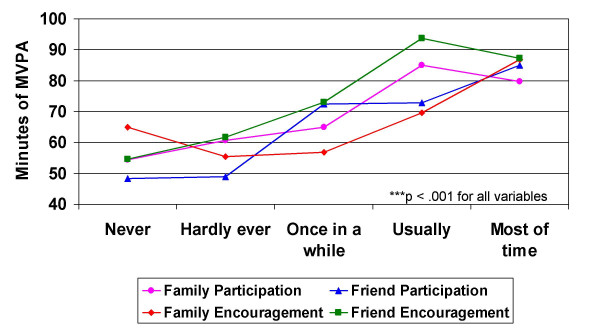
Mean daily minutes of moderate-to-vigorous physical activity (MVPA) by frequency of family & friend participation in and encouragement for physical activity (n = 667).

Overall, mean daily minutes of MVPA increased with frequency of family and friend social support indicators (Figure 1). Although all bivariate correlations between the social support variables and moderate-vigorous physical activity were positive and statistically significant (p < .05), they were generally low (Table 2). The correlation between BMI and MVPA was negative, as expected, but was not statistically significant. The linear combination of social support measures and the BMI and ethnicity covariates was significantly related to daily minutes of MVPA [*F*(7, 634) = 9.84, p < .001]. The sample multiple correlation coefficient was .31, indicating that 9.8% of the variance of MVPA can be accounted for by the linear combination of social support measures and BMI and ethnicity. Results of the full model revealed that the partial correlations were significant for all social support variables with the exception of family participation (Table 2).

**Table 2:** Bivariate, partial correlations and standardized regression coefficients (β) of family and friend social support variables with daily minutes of moderate-vigorous and vigorous physical activity among female middle school students, IMPACT Baseline Study, 2001

	Moderate-Vigorous Physical Activity			Vigorous Physical Activity		
		
Predictors	Correlation between predictor and MVPA	Correlation between predictor and MVPA after controlling for all other predictors	β	Correlation between predictor and VPA	Correlation between predictor and VPA after controlling for all predictors	β
Family Participation^a^	0.17***	0.07	0.08	0.08*	0.05	0.05
Family Encouragement^b^	0.18***	0.11**	0.13**	0.08	<0.001	<0.001
Family Encourage.(squared)^c^	0.01	0.11**	0.12**	N/A	N/A	N/A
Friend Participation^a^	0.21***	0.10**	0.11**	0.10**	0.03	0.04
Friend Encouragement^b^	0.25***	0.12**	0.14**	0.16***	0.11**	0.13**
Body Mass Index	-0.01	-0.01	-0.01	0.05	0.04	0.04
Ethnicity^d^	0.01	0.04	0.04	0.09*	0.11**	0.11**

Abbreviations: Family Encourage., family encouragement; MVPA, moderate-to-vigorous physical activity; VPA, vigorous physical activity; N/A, not applicable.

^a^Family Participation/Friend Participation: frequency that family or friends did physical activities with student during past month based on 5-point scale from "never" to "most of the time"

^b^Family Encouragement/Friend Encouragment: frequency that family or friends encouraged student to be physically active during the past month based on 5-point scale from "never" to "most of the time"

^c^Family encouragement squared = family encouragement centered (mean) and squared.

^d^Ethnicity = White, Hispanic, Other

* p < .05, ** p < .01, *** p < .001

### Vigorous Physical Activity (VPA)

**Figure 2:**
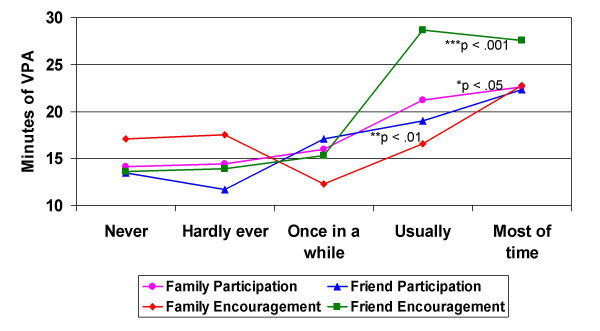
Mean daily minutes of vigorous physical activity (VPA) by frequency of family & friend participation in and encouragement for physical activity (n = 664).

Students reporting higher levels of family and friend social support also reported higher mean daily minutes of VPA as compared with students with the lowest frequency of social support (Figure 2). With the exception of family encouragement, all bivariate correlations between social support variables and VPA were significant (Table 2). Although the linear combination of the social support variables and covariates was significantly related to VPA [*F*(6, 635) = 4.51, p < .001], the full model accounted for a smaller amount of variance (R^2 ^= .04) compared with the model with MVPA. In contrast to the MVPA, only friend encouragement was found to be significant in the full model after controlling for each social support variable and the BMI and ethnicity covariates (p = .004).

### Sedentary behaviors

**Figure 3:**
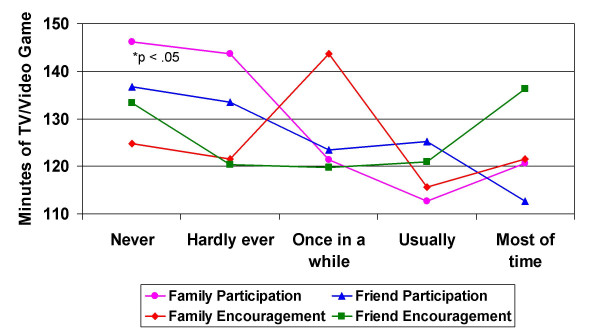
Mean daily minutes of combined sedentary behavior (TV/Video Game) by frequency of family & friend participation in and encouragement for physical activity (n = 706).

With the exception of the friend encouragement variable, mean daily minutes of the combined sedentary behaviors of TV-video viewing and computer-video game playing were lower as the frequency of family and friend physical activity participation increased (Figure 3). Although all bivariate correlations between the social support variables and sedentary behavior were negative, family participation in physical activity was the only statistically significant social support variable (Table 3). The linear combination of social support measures was found to be significantly related to daily minutes of combined sedentary behavior [*F*(6, 670) = 5.10, p < .001]. However, only a small amount of the variance of sedentary behavior minutes can be accounted for by the linear combination of the social support variables [R^2 ^= .04]. Family participation was the only statistically significant variable in the full model after controlling for the other social support variables and the BMI and ethnicity covariates. The bivariate correlation between BMI and combined sedentary behavior minutes was positive, as expected, and BMI was significantly correlated with combined sedentary behavior in the full model.

**Table 3:** Bivariate, partial correlations and standardized regression coefficients (β) of family and friend social support variables with daily minutes of combined sedentary behavior^a ^among female middle school students, IMPACT Baseline Study, 2001

	Combined Sedentary Behavior		
	
Predictors	Correlation between predictor and sed. behavior	Correlation between predictor and sed. behavior after controlling for all other predictors	β
Family Participation^b^	-0.11*	-0.08*	-0.08*
Family Encouragement^c^	-0.04	-0.02	-0.02
Friend Participation^b^	-0.08	-0.05	-0.05
Friend Encouragement^c^	-0.01	0.05	0.05
Body Mass Index	0.14***	0.12**	0.12**
Ethnicity^d^	0.12**	0.11**	0.11**

Abbreviations: P.A., physical activity; sed., sedentary

^a^Combined Sedentary Behavior is the sum of daily T.V.-video viewing minutes and daily computer-video game playing minutes

^b^Family Participation/Friend Participation: frequency that family or friends did physical activities with student during past month based on 5-point scale from "never" to "most of the time"

^c^Family Encouragement/Friend Encouragment: frequency that family or friends encouraged student to be physically active during the past month based on 5-point scale from "never" to "most of the time"

^d^Ethnicity = White, Hispanic, Other

* p < .05, ** p < .01, *** p < .001

## Discussion

This study examined the association of two types of social support (physical activity social participation and social encouragement) and two sources of social support (from family and friends) with daily minutes of MVPA, VPA, and sedentary behaviors among 6th-grade girls living in Texas. While the correlations were modest, we found that family and friend physical activity participation and encouragement were positively related to higher daily minutes of MVPA in bivariate analyses and that all but family participation maintained significance in the regression analysis of the full model. Although we found family participation, friend participation, and friend encouragement to be positively related to daily minutes of VPA in bivariate analyses, friend encouragement was the only variable significantly related to VPA in the regression analysis. For sedentary behaviors, family physical activity participation was the only social support variable significantly and inversely related to combined minutes of TV-video viewing and computer-video game playing in both bivariate and multivariable analyses. These findings suggest that the frequency of friend physical activity participation and friend and family physical activity encouragement may play important roles in the physical activity levels of girls in early adolescence, whereas the frequency of family participation in physical activity may be important for reducing the amount of time spent in sedentary behaviors.

### Type of social support

Although several studies have indicated the importance of various types of social support for physical activity among adolescents [12,15], we found that both physical activity participation and encouragement were important for MVPA but that encouragement had the strongest correlation with VPA. These differences may relate to the type of physical activity, in which MVPA such as walking or dancing may be more amenable to social participation, and vigorous physical activities that are more individually focused and perceived to be of greater intensity – such as running or biking – may be more influenced by encouragement.

Conversely, our finding that family participation in physical activity had the strongest negative correlation with sedentary minutes among the social support variables suggests that family participation in physical activity may be more important than encouragement for reducing sedentary behavior. One explanation for the significant inverse association observed between family physical activity participation and time spent in sedentary behaviors is the potential influence of other types of social support on physical activity behavior that stem from family physical activity participation, such as instrumental and observational social support. Transportation to a recreational area where the family carries out physical activity, for example, is a form of instrumental support that may increase a child's opportunities for physical activity while reducing his/her time spent in sedentary behavior. As some studies have found parents' and children's inactivity [26,27] and television behavior [28] to be similar, our findings of the importance of participation over encouragement may also suggest that observational support via family modeling of physically active behaviors may be important for reducing children's sedentary behaviors.

### Source of social support

Although we found that both friend and family support were significantly correlated with girls' physical activity levels, the significant correlations between both friend social support indicators with MVPA as well as friend encouragement with VPA may suggest that friends play a more important role in influencing physical activity levels of adolescent girls. Sallis et al. [15] found peer support to be significantly associated with VPA in multiple regression analyses among female girls from Massachusetts in grades 7 through 9. Prochaska et al. [13] found parental and peer influences were significant correlates of self-reported physical activity among middle school students in California but found peer support to be the strongest correlate in a regression model. Most recently, Duncan et al. [29] found friend social support to be most highly related to physical activity among youth between the ages of 10 and 14 in a model that included parental and sibling sources of support. These findings, along with the current study's findings, suggest that peers may take a prominent role in influencing physical activity among adolescent girls.

Family physical activity participation was found to have the strongest negative correlation with combined sedentary minutes of TV-video viewing and computer-video game playing, which may be explained in part by the contextual nature of these sedentary behaviors within the home environment. Woodard and Gridina [11] report that 48% of households with U.S. children between the ages of 2 and 18 have all four of the principal media staples (a television, a VCR, video game equipment, and a computer). Roberts et al. [30] found that 65% of children between the ages of 8 and 18 years had a television set in their bedroom and that children spend an average of 6 hours and 32 minutes per day with various media. The greater access to media within the home environment and the large amount of time spent with media suggest the importance of the home environment for these types of behaviors. Underscoring the important relation between the family and child's physical activity levels, a review of 54 studies of potential correlates of physical activity among adolescents found measures of sibling physical activity and parental support – including direct parental help in physical activity – to be consistently related to adolescent physical activity [12]. Trost and colleagues [31] found parental support, a composite variable that included parental participation in physical activity with child and parental encouragement, to directly influence child physical activity and to mediate the relation between parental physical activity and child physical activity. The inverse relation between time spent in sedentary pursuits and higher family physical activity participation found in our study along with the strong body of evidence for the role of family support with adolescent physical activity suggest that families may exert important influence over an adolescent's available time within the home environment and that families are an important target for reducing sedentary behavior.

### Body mass index, sedentary behavior, and physical activity

Our finding of an association between BMI and television viewing is consistent with findings from several large-scale national studies on television and BMI of children and adolescents [32-34]. Although we also found an association between BMI and computer-video game playing, the role of video game playing in childhood obesity has received less attention than television viewing, with studies reporting mixed results [35-37]. Additional research is needed to determine the association between obesity and video game playing among children and adolescents.

Several hypotheses exist regarding the association of obesity and sedentary behaviors, such as: a.) sedentary behaviors displace time that could be spent in physical activity [9], b.) long periods of inactivity and snacking while engaged in sedentary behaviors contribute to obesity [9], and c.) snacking and concomitant excess energy consumption are encouraged by television ads targeted at high energy, low nutrient foods [38,39]. As this study did not find an association between BMI and physical activity, the latter hypotheses may be relevant for understanding the linear association found between BMI and sedentary behavior. An interesting finding of this study was that girls in the highest BMI percentile group reported the highest number of vigorous activity minutes. While a social desirability reporting bias cannot be ruled out in interpreting this finding, alternative explanations may be that: a.) higher BMI girls are engaging in more vigorous physical activity with the aim of reducing their weight, or b.) girls engaged in more VPA may be more active overall, leading to comparatively more muscle tissue, and thus, the typical BMI-body fat associations are not as relevant.

Exploring the association of BMI and social support is important as some researchers have indicated that overweight adolescents may be more socially isolated [40] and others have found overweight children to report lower levels of social support for physical activity [41,42]. Our findings did not demonstrate differences in the two social support indicators examined by BMI classification. Further research is needed to explore the association between BMI and social support.

### Strengths & limitations

In addition to contributing to the literature on social support and physical activity, this study is among the first to examine the relation between social support indicators and sedentary behaviors among middle school girls. A second important strength of this study was the administration of the SAPAC on three separate days – including one weekend day, which provides a more stable estimate of daily minutes of physical activity, TV-video viewing and computer-video game playing. These strengths notwithstanding, some limitations should be considered in interpreting the study's findings.

Reliance on self-report instruments to measure both social support and physical activity is a potential limitation that may have led to overestimation of the correlations observed in this study. Prochaska et al. [13], in their study of Californian middle school students, found that correlations between self-reported social support and physical activity were significant but correlations between self-reported social support and monitored physical activity were not. Possible explanations for these findings provided by Prochaska and colleagues include a shared method variance based on the self-report measures' common mode of assessment as well as increased measurement of incidental physical activity from activity monitors as compared to intentional physical activities measured by the self-report instrument.

This study was also limited to a secondary analysis of two types of social support from two sources that were based on one-item measures. As such, the social support indicators do not capture the various conceptualizations of social support as related to adolescent physical activity or the richness of social ties that surround children, which could have led to an underestimation of the relation between the social support indicators and physical activity/sedentary behavior found in this study. Because this secondary analysis was based on data not specifically designed to investigate social support and sedentary behaviors, we were not able to examine social support that directly targets decreased sedentary behavior. Other limitations include the relative ethnic homogeneity of the sample, which reduces the generalizability of the results to ethnically diverse adolescent girls, and the cross-sectional study design, which limits inferences on the causality of the relation between the social support variables and the physical activity and sedentary behavior outcomes. This final limitation is worth highlighting as it is possible that students who were more physically active actually promoted physical activity among their friends and family and that those who were more sedentary negatively impacted their family's participation in physical activity.

## Conclusion

Our findings lend support to the importance of family and friend social support for physical activity among adolescent girls but suggest that the source and type of social support may differ for physical activity and sedentary behavior. While both peer and family relationships were found to be significantly associated with MVPA among adolescent girls, our findings indicate that peer relationships may be more important for increased vigorous physical activity and family relationships may be more important for decreased sedentary behaviors such as TV-video viewing and computer-video game playing. In terms of the types of social support, our findings suggest that both social participation and encouragement are important for physical activity, but that family participation may be more important than encouragement for decreasing sedentary behaviors.

Future research should examine the relative influence of additional and more specific types of social support, such as instrumental support as related to transportation to physical activities. The development of multi-item scales with psychometric properties that distinguish among the various types of social support as well as inclusion of items that provide more range and specificity on the sources of social support (e.g., siblings, mother and father vs. family) would also strengthen the study of social support and physical activity/sedentary behavior. Lastly, future research should explore the causal and potentially reciprocal relation of social influences and physical activity among adolescents.

## Competing interests

The author(s) declare that they have no competing interests.

## Authors' contributions

AS performed the statistical analysis of the data and took the primary role in drafting the manuscript. SK guided the strategy of the analysis of the data, assisted with the interpretation of the results, contributed to the writing of the methods section, and provided critical review of the manuscript. DH contributed to the writing of the methods section and provided critical review of the manuscript. AS, SK and DH conceived of the study. DH was the Principal Investigator and SK was a co-Investigator of the original IMPACT study. All authors read and approved the final manuscript.
